# The effect of vision impairment on dynamic balance

**DOI:** 10.1186/1757-1146-8-S1-A6

**Published:** 2015-04-20

**Authors:** Richard Collings, Joanne Paton, Sam Glasser, Jonathan Marsden

**Affiliations:** 1 BEUP, Torbay and Southern Devon Health and Care NHS Trust, Devon, UK; 2 BEUP, School of Health Professions, Faculty of Health and Human Sciences, Plymouth University, Plymouth, UK

## Aim

The aim of this study was to present the effect of visual acuity impairment on dynamic balance using an in-shoe pressure measurement system.

## Relevance/Impact

The control of human gait and the maintenance of balance depend upon the complex integration of visual, vestibular and somatosensory information. Dysfunction of any of these components can result in deficits in the body’s ability to maintain equilibrium of the centre of mass by counteracting the constant destabilising forces that challenge it. The role of vision in the control of balance is well documented. Vision can improve bipedal upright stability during standing and locomotion as part of the integrated sensory feedback system. Alternatively vision impairment has been demonstrated as reducing postural stability. Postural stability is traditionally evaluated by the motion associated with changes in Centre-of-Pressure (CoP) during quiet standing. CoP measures have been shown to high reliability and clinical relevance when assessing postural stability. However there are few studies that have assessed the effectiveness of these measures for dynamic stability.

## Methods

An in-shoe pressure system (FScan, Tekscan, UK) was used to measure CoP values during walking gait for 15 asymptomatic subjects along a 5 metre walkway. Walking was assessed under normal and altered visual conditions using vision impairment goggles (BAC 0.08-0.15, DAI Impairment goggles, UK).

Outcome measures used to assess dynamic stability

1) Rate of medial-lateral progression of CoP (mm/s) in 50-100% stance phase of

2) Extent of medial-lateral excursion of CoP (mm) in 50-100% stance phase of gait

3) CoP position (mm) in 50-100% stance phase of gait

## Results

A two-tailed paired t-test analysis of the means demonstrated that impaired vision resulted in an increase in the variability of the CoP position (t=-3.6 P<0.005) and that there was an increase in the rate of medial-lateral CoP motion with impaired vision t(14)=2.63 p<0.019. Further, the CoP was 0.4 mm (±0.61mm) more medial during reduced vision conditions (t=2.5 P<0.05).

## Discussion

In the present study there were significant changes in gait variability, as measured by the CoP indicators in the medial–lateral plane when vision was impaired. Other studies report similar findings that show impaired / perturbed vision increased sway and reduced balance in quiet standing. This may suggest that our proxy measures are suitable for assessing dynamic stability for this particular population. Our findings indicate that an in-shoe-pressure measurement system may be useful in the clinical assessment dynamic postural instability for those with visual impairment. Measuring changes in COP parameters during quiet standing may not fully reflect changes in CoP parameters during gait. The ability to capture CoP variability as a measure of balance during gait could have greater clinical relevance then more traditional static methods. Further work on testing the reliability of these measures and results in elderly populations would be advantageous to the development of strategies to reduce the impact of visual impairment on dynamic balance to reduce falls risk.

